# Potent RBD-specific neutralizing rabbit monoclonal antibodies recognize emerging SARS-CoV-2 variants elicited by DNA prime-protein boost vaccination

**DOI:** 10.1080/22221751.2021.1942227

**Published:** 2021-07-08

**Authors:** Yuxin Chen, Liguo Zhu, Weijin Huang, Xin Tong, Hai Wu, Yue Tao, Bei Tong, Haibin Huang, Jiachen Chen, Xiangan Zhao, Yang Lou, Chao Wu

**Affiliations:** a Department of Laboratory Medicine, Nanjing Drum Tower Hospital, Nanjing University Medical School, Nanjing, People’s Republic of China; b Jiangsu Provincial Center for Disease Control and Prevention, Nanjing, People’s Republic of China; c Division of HIV/AIDS and Sex-Transmitted Virus Vaccines, Institute for Biological Product Control, National Institute for Food and Drug Control, Beijing, People’s Republic of China; d Department of Infectious Diseases, Nanjing Drum Tower Hospital, Nanjing University Medical School, Nanjing, People’s Republic of China; e Yurogen Biosystem LLC, Worcester, MA, USA; f Institute of Botany, Jiangsu Province and Chinese Academy of Sciences, Nanjing, People’s Republic of China; g Department of Gastroenterology, Northern Jiangsu People’s Hospital, Clinical Medical College of Yangzhou University, Yangzhou, People’s Republic of China

**Keywords:** SARS-CoV-2, rabbit mAb, vaccine, variant of concerns

## Abstract

Global concerns arose as the emerged and rapidly spreading SARS-CoV-2 variants might escape host immunity induced by vaccination. In this study, a heterologous prime-boost immunization strategy for COVID-19 was designed to prime with a DNA vaccine encoding wild type (WT) spike protein receptor-binding domain (RBD) followed by S1 protein-based vaccine in rabbits. Four vaccine-elicited rabbit monoclonal antibodies (RmAbs), including 1H1, 9H1, 7G5, and 5E1, were isolated for biophysical property, neutralization potency and sequence analysis. All RmAbs recognized RBD or S1 protein with K_D_ in the low nM or sub nM range. 1H1 and 9H1, but neither 7G5 nor 5E1, can bind to all RBD protein variants derived from B.1.351. All four RmAbs were able to neutralize wild type (WT) SARS-CoV-2 strain in pseudovirus assay, and 1H1 and 9H1 could neutralize the SARS-CoV-2 WT authentic virus with IC_50_ values of 0.136 and 0.026 μg/mL, respectively. Notably, 1H1 was able to neutralize all 6 emerging SARS-CoV-2 variants tested including D614G, B.1.1.7, B.1.429, P.1, B.1.526, and B.1.351 variants, and 5E1 could neutralize against the above 5 variants except P.1. Epitope binning analysis revealed that 9H1, 5E1 and 1H1 recognized distinct epitopes, while 9H1 and 7G5 may have overlapping but not identical epitope. In conclusion, DNA priming protein boost vaccination was an effective strategy to induce RmAbs with potent neutralization capability against not only SARS-CoV-2 WT strain but also emergent variants, which may provide a new avenue for effective therapeutics and point-of-care diagnostic measures.

## Introduction

The coronavirus disease 2019 (COVID-19) caused by the infection of severe acute respiratory syndrome coronavirus 2 (SARS-CoV-2) was initially identified in December 2019 [1]. The rapid spread of COVID-19 has resulted in more than 156 million confirmed cases and caused around 3.2 million deaths, as of May 8, 2021. The ongoing pandemic continues posing an unprecedented global threat to public health systems. Several promising therapeutic agents including antiviral agents, immunomodulators, glucocorticoids, and convalescent plasma, have been evaluated for treating the COVID-19, generating varying results. However, there are limited approved therapeutic agents against the SARS-CoV-2 available to the general public. SARS-CoV-2 uses the envelope spike (S) glycoprotein, composed of two subunits S1 and S2, to mediate host cell entry. S1 facilitates viral attachment to a cell surface receptor, angiotensin-converting enzyme (ACE2) [2], via its receptor-binding domain (RBD), while S2 is essential for membrane fusion [3]. Disruption of the RBD-ACE2 interaction can block SARS-CoV-2 cell entry, presenting a valid mechanism of clinical interventions.

Due to such an essential role of spike protein during viral entry, recent emerging SARS-CoV-2 variants bearing multiple mutations within spike protein have rasied serious concerns. The recent circulating variants include the D614G variant, the B.1.1.7 variant in the United Kingdom, the B.1.351 variant in South Africa, the P.1 variant in Brazil, the B.1.429 variant in California, and the B.1.526 variant in New York. D614G is one of the earliest variants that rapidly emerged and became globally dominant, while convalescent sera demonstrated efficient cross-neutralization for both wild-type strain and D614G variants [4]. B.1.1.7 contains D614G and N510Y in the RBD region, with only slightly neutralization resistance by convalescent individuals and vaccine recipients [5, 6]. The B.1.429 variant, a lineage recently emerged in California, United States, contains 4 missense mutations in spike including a single L452R RBD mutation [7]. The B.1.351 variant (also called 501Y.V2) is one of the greatest variants of concern (VOCs), which was first reported in South Africa and rapidly spreading. B.1.351 bears three RBD mutations (K417N, E484K, and N501Y), in addition to several mutations outside of RBD, leading to substantial or complete loss of neutralization potency from humoral immunity elicited by natural infection and vaccination [7–10]. The P.1 strain in Brazil harboring three RBD mutation has also been demonstrated with effectively escaped neutralization [11, 12]. The B.1.526 variant, another variant currently spreading alarmingly in New York, United States, shares the prevalent D614G and E484K mutation in the RBD region, raising the concerns of escaped immunity for neutralization activity [13]. These fast-spreading SARS-CoV-2 variants call for enhanced viral surveillance and efficacy assessment of currently authorized vaccines, therapeutic monoclonal antibodies (mAbs) and convalescent sera against the emerging variants.

Passive administration of monoclonal antibodies (mAbs) has become one of the essential therapeutic agents [14] especially to infectious diseases [15, 16]. A few potent SARS-CoV-2 specific neutralizing antibodies have been identified from infected COVID-19 patients [17–23], immunized mice [24], and llama [25]. Some of these neutralizing antibodies such as bamlanivimab, etesevimab and casirivimab have received Emergency Use Authorization (EUA) by respective regulatory agencies [26]. Accumulating evidence suggested that administration of neutralizing antibodies at the early infection stage could minimize hospitalization and decreases symptom severity among non-hospitalized patients [27], further substantiating neutralizing monoclonal antibodies as valid treatment agents in COVID-19 patients.

Since the rabbit immune system has a unique ontogeny of B cells that is different from mice and other rodents, immunized rabbits usually generate highly diverse antibody repertoires with superior diversity, affinity, and specificity. Improved access to rabbit antibody repertoires by high throughput technologies, such as hybridoma [28], single B cells sorting approach [29, 30] and mass spectrometry [31], has fueled the increased application of rabbit mAbs. Therefore, rabbit monoclonal antibodies (RmAbs) have been widely used for clinical diagnostic and therapeutic applications. Recently, the inhibitor of VEGF-A, Brolucizumab, was the first humanized rabbit ScFv that received FDA approval for patients with wet age-related macular degeneration (AMD) in 2019 [32].

Heterologous prime-boost vaccination strategies using DNA priming vaccine have been adopted in early clinical trials in the context of infectious diseases including HIV [28, 33, 34], influenza [35–37] and malaria [38], eliciting robust and balanced humoral responses and cellular responses. DNA immunization can effectively stimulate both the innate and adaptive immunities to generate high levels of antigen-specific antibody responses, producing functional potent mAbs, in mouse, rabbit and human models [39]. A DNA vaccine (INO-4800) targeting the full-length spike antigen of SARS-CoV-2 has demonstrated an excellent safe and tolerable file, which is immunogenic in 100% vaccinated subjects, as indicated by results from phase 1 clinical trial [40]. A recent COVID-19 vaccine study also suggested that employing a heterologous prime-boost strategy might break the protective immune response bottleneck, inducing balanced neutralizing antibody responses and T cell responses [41].

To analyze the antibody repertoire generated by DNA priming coupled with a protein boost, we utilized SARS-CoV-2 spike protein S1 subunit as the bait to isolate S1-specific single memory B cells from the splenocytes of immunized rabbits. RmAbs were expressed and purified for functional assays. Our data identified at least four highly potent neutralizing rabbit mAbs specific to RBD revealed by either wild-type authentic virus or pseudovirus neutralization assay, indicating a successful vaccination strategy of DNA prime followed by protein boost. More importantly, RmAb 1H1 was able to neutralize all 6 emerging SARS-CoV-2 variants tested including P.1 and B.1.351 variants, two variants of greatest concern, providing a robust basis for developing new therapeutics. Our study sheds light on the development of antibody-based therapeutic treatments and rational vaccine design against the SARS-CoV-2.

## Materials and methods

### Construction of SARS-CoV-2 DNA vaccine encoding RBD domain and DNA bullet preparation

The codon-optimized RBD region, corresponding to the genomic positions 22,553 to 23,312 bp in SARS-CoV-2 isolate Wuhan-Hu-1 (GenBank: MN908947.3), was cloned into the pcDNA3.4 expression vector. The expression construct was propagated in DH5α strain of *Escherichia coli* and purified using the Qiagen Plasmid Mega kit (Cat no. 10023). 36 μg purified expression construct were coated to 100 μL of 100 mg/ml gold powder (Alpha Aesar, Catalog No. 39817) that was precoated with 100 mg/ml spermidine (Sigma, Catalog No. S2626). DNA-coating to gold was facilitated by slowly dripping 200 ul 2.5 M CaCl_2_ to the DNA mixed with gold powder, which was washed in absolute ethanol before loading to bullet tubing mounted to bullet maker (Scientz Scientific). Bullet tubing loaded with gold powder was dried by slow N_2_ flow at 0.1 MPa for 10 min, and the dried bullet tubing was cut into DNA bullet.

### Recombinant SARS-CoV-2 protein

Recombinant SARS-CoV-2 S1 protein fused with a Fc tag at C terminal was acquired from Kactus Biosystems, Shanghai (Cat no: COV-VM5S1) for ELISA. Recombinant RBD protein (Kactus Biosystems, Cat no: COV-VM4BD) and SARS-CoV-2 S1 protein fused His tag (Kactus Biosystems, Cat no: COV-VM4S1) were obtained for analyzing ELISA and immunization. Recombinant SARS-CoV-2 ECD protein (Genscript, Cat no: Z03481) and the related RBD variants (RBD N501Y, RBD K417N, RBD E484K, RBD N501Y/K417N/E484K) were used for ELISA binding assay.

### Rabbit immunization

New Zealand White rabbits (4–6 weeks of age) were purchased and housed in the animal facility. All the procedures were carried out by following animal research guidelines and approved by IACUC (Abclonal, China). The SARS-CoV-2 RBD DNA bullets were loaded into the bullet magazine of the SJ-500 gene gun (Scientz Scientific). The SJ-500 gene gun was fired by 4 MPa helium gas to inject DNA-coated gold powder subcutaneously at shaved abdomen skin (36 μg/immunization) of rabbits. The DNA immunization was performed three times on each rabbit at days 0, 7, and 21. Then, the rabbits were boosted with 100 μg SARS-CoV-2 S1 protein emulsified in incomplete Freund’s adjuvant (IFA) twice at day 35 and day 49 via intramuscular (i.m.) route. Two weeks later, the rabbits were boosted subcutaneously (s.c.) with 200 μg S1 protein. Pre and post-immunization sera were collected on days 0, 14, 28, 42 and 69, respectively.

### ELISA for characterization of immunized rabbit sera and monoclonal antibody

The enzyme-linked immunosorbent assay (ELISA) was performed as previously described [42]. Briefly, 96-well microtiter plates (Corning, Cat no: 9018) were coated with 100 μL of 1 μg/mL ectodomain of spike protein (ECD), S1 protein, RBD protein, RBD variants protein (RBD E484K, RBD K417N, RBD N501Y, RBD N501Y/K417N/E484K), in coating buffer (pH 9.6) overnight at 4°C, respectively. Plates were washed three times in washing buffer (1× PBS supplemented with 0.05% Tween-20 (Sigma, Cat no: P9416)) and blocked with 200 μL blocking buffer (1 × 5% nonfat milk in 1× PBS). Then the plates were incubated with serial diluted rabbit serum samples or monoclonal antibody. After 1-hour incubation at room temperature, plates were washed five times with washing buffer and incubated with anti-rabbit IgG conjugated with HRP (Jackson ImmunoResearch, Cat no: 111-035-045) in blocking buffer at 1:5000 dilution. Plates were washed five times with washing buffer and developed by adding 25 μL TMB substrate (Moss INS, Cat no: TMBHK-1000) for 3 min in the dark at room temperature. Subsequently, the colorimetric reaction of TMB substrate was stopped with 20 μL 1 M H_2_SO_4_. Optical density (OD) values at 450 nm and 630 nm were measured by Epoch microplate spectrophotometer (Biotek, USA). The final value was obtained using OD_450_ subtracted by OD_630_. The serum titer was calculated as the maximum dilution where the diluted serum produces OD450 reading of 2-fold or above than that of the control samples.

### Isolation of S1-specific single B cells from rabbit splenocytes by fluorescence-activated cell sorting (FACS)

The spleen was harvested from the rabbit with higher titers against the SARS-CoV-2 S1 and RBD domains. Fresh single splenocytes were isolated and cultured overnight in a specialized B cell medium specialized by Yurogen Biosystems (Wuhan, China). A fresh single-cell suspension of solenocytes in PBS supplemented with 2% FBS and 1mM EDTA was prepared before single-cell sorting. Splenocytes were processed using the single B cell SMab® platform [43] at Yurogen Biosystems and sorted one cell per well on a FACS Aria II (BD Biosciences, USA) into 96-well plates. S1-specific primary B cells were cultured in proprietary rabbit B cell complete medium (Yurogen, China) for 10–14 days at 37°C with 5% CO_2_.

### Generation of rabbit mAbs

At the end of primary B cell culture, S1-recognizing B-cell clones were identified by screening of primary B cell culture supernatants against RBD or S1 by direct ELISA. Positive B-cell clones were determined by OD450nm values more than 5-fold over background noise. The variable region of IgG heavy chain and light chain from top positive clones was recovered by RT-PCR. The full-length IgG heavy and light chains of each clone were co-transfected into HEK293T cells. The supernatants containing recombinant rabbit IgG from transfected HEK293T were screened for their specificity to S1 and RBD by ELISA. The PCR fragments of variable regions of selected clones were cloned into pcDNA3.4 vector for antibody expression in HEK293F cells. Recombinant rabbit mAbs were purified using protein A chromatography for further characterization.

### SARS-CoV-2 pseudotyped virus neutralization assays

Pseudovirus neutralization assay was performed as previously described with minor modifications [44]. Briefly, SARS-CoV-2 pseudoviruses were prepared using VSV G pseudotyped viruses (G*ΔG-VSV) that package the expression cassette for firefly luciferase instead of VSV-G in the VSV genome. The VSV-based SARS-CoV-2 pseudoviruses contained wild-type, D614G, B.1.351, B.1.1.7, P.1, B.1.429 and B.1.526 variants spike protein. SARS-CoV-2 pseudoviruses were pre-incubated with mAbs starting from 10 at 37°C for one hour, together with the pseudovirus control and cell control wells. The 96-well plates were seeded with 100 μL of freshly trypsinized Huh7 cells (2 × 10^4^ cells/well). After 24 h of incubation in a 5% CO_2_ environment at 37°C, the luminescence was measured using luciferase substrate (One-GloTM Luciferase assay system, Promega, E6120). Half-maximal inhibitory concentrations (IC_50_) of the RmAbs were determined by the concentration of RmAb at which the relative light units (RLUs) were reduced by 50% compared to the virus control wells after subtraction of background RLUs of the control groups with cells only. The IC_50_ values were calculated using nonlinear regression in GraphPad Prism 9.0.

### Authentic neutralization assay against live SARS-CoV-2 viruses

The neutralization activity of mAbs against live SARS-CoV-2 was performed in a certified biosafety level 3 laboratory. Live SARS-CoV-2 strain was previously isolated from a nasopharyngeal swab of an infected patient from Jiangsu province, China. Briefly, Vero cells were seeded in 24-well plates (200,000 cells/well) and incubated for approximately 16 h until 90–100% confluent. Serial 3-fold dilution of mAbs 1H1 and 9H1 prepared in DMEM containing 2% FBS was then mixed with titrated virus in a 1:1 (vol/vol) ratio to generate a mixture containing 100 focus-forming units (PFU)/ml of viruses, followed by incubation at 37°C for 1 h. The complexes of mAb and virus were added to wells of 24-well plates of Vero cell monolayers in duplicate and then incubated at 37°C for 1 h. The mixtures were removed and cells were overlaid with 1% low-melting-point agarose (Promega) in DMEM containing 2% FBS. After incubation at 37°C for 3 days, the cells were fixed with 4% formaldehyde and stained with 0.2% crystal violet solution (Sigma). SARS-CoV-2-infected cell foci were visualized through the plaque numbers. The 50% inhibitory concentration (IC_50_) of mAb was defined as the concentration of antibody (μg/mL) resulting in a 50% reduction relative to the total number of plaques counted without antibody [45].

### Surface plasmon resonance analysis

Surface plasmon resonance (SPR) kinetic analysis was conducted using a BIAcore 3000 instrument with Protein A sensor chip (GE Healthcare, USA). All experiments were performed at 25°C at a flow rate of 40 μL/min. The running buffer was degassed PBS with 0.005% Tween-20. Channel 1 was loaded with a reference antibody without specific binding to the antigens used, and channel 2, 3, and 4 were loaded with the antibody candidates. Typically, 2 μg/mL of antibody and a quick injection for 20–30 s yielded ∼150–250 response units (RU) of antibody coupling with high reproducibility. Antigen (S1 or RBD protein) was then injected over all the channel surfaces for 5 min for an association phase followed by a 10-minute dissociation phase by buffer rinse. Multiple association/dissociation cycles were performed using antigen dilution series in the range of 1.2–100 nM, as well as a blank buffer. At the end of each cycle, regeneration was performed by a 30-second injection of glycine buffer (pH 2.0, 10 mM) and antibodies were loaded in each channel again. The kinetic curves were double reference subtracted and analyzed to calculate association rate constant, dissociation rate constant, and affinity constants using BIA evaluation 3.2 and 1:1 Langmuir model.

### Epitope binning assay

All epitope binning assays were performed on the Gator system (Probe Life, USA) and using the Tandem model for epitope binning of mAbs. Briefly, the anti-His-tag sensor (Probe life) was pre-wetted in Q buffer (Probe Life), then immobilize 0.5 μg/ml of His-tag SARS-COV2-S1 protein (Kactus Biosystems, Cat. No.COV-VM4S1) to the sensors. The antigen-loaded sensor captured 5 μg/ml of the first rabbit monoclonal antibodies for about 300 s, then the loaded sensor captured 1 μg/ml of the second monoclonal antibodies for about 600 s.

### ELISA assay for ACE2 receptor blocking

Half maximal effective concentration (EC50) of RBD binding to ACE2 was determined using ELISA. Recombinant ACE2 (Kactus Biosystems, Cat. No. ACE-HM501) was coated at 1 μg/ml on an ELISA plate. B cell supernatant from positive clones with 4 points of 3-fold serial dilution was preincubated with RBD domain protein diluted at EC50 for 1 h at room temperature. B cell supernatant-RBD premix was then deposited to ACE2-coated ELISA plate for 1 h at room temperature.

### Analysis of rabbit mAb heavy chain and light chain sequences

Sequencing analysis was conducted with the heavy-chain and light-chain genes of rabbit mAbs. Germline V(D)J gene annotation was performed using IMGT/V-QUEST[46]. ClustalW was used and the multiple sequence alignments were performed to construct phylogenetic trees using maximum-likelihood methods. Antibody germline usage, mutation frequency and CDR3 length were determined by IMGT/V-QUEST [47].

## Results

### Rabbit immunization with RBD DNA prime followed by S1 protein boost

**Figure 1.**
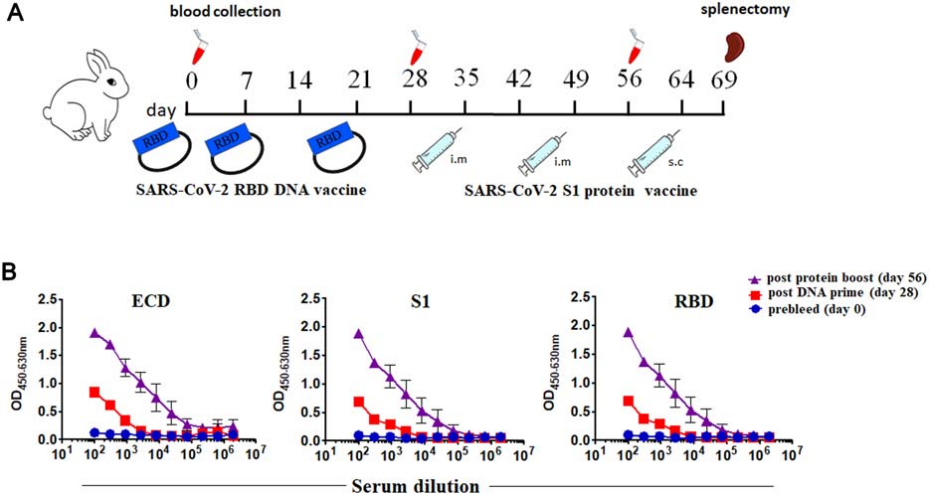
Immunization strategy of DNA prime followed by protein boost successfully induced SARS-CoV-2 specific and neutralizing antibody responses. (A) Two rabbits received three doses of DNA vaccine encoding the SARS-CoV-2 RBD on day 0, day 7 and day 21, and were further boosted with two doses of the SARS-CoV-2 S1 protein emulsified with incomplete Freund’s adjuvant (IFA) on day 35 and day 49. Sera were collected on day 0, day 28, and day 56 for immunoassays. (B) The serological IgG titer specific to RBD, S1 and ECD protein from naive rabbits (day 0) and immunized rabbits (days 28 and 56).

To produce high-quality SARS-CoV-2 neutralizing antibodies, two female New Zealand white rabbits received 36 μg DNA vaccine encoding SARS-CoV-2 RBD region at day 0, day 7 and day 21. 14 days after primary immunization, rabbits were boosted with two doses of 100 μg recombinant SARS-CoV-2 S1 protein emulsified with incomplete Freund’s adjuvant (IFA) via intramuscular injection on day 35 and day 49. Sera were collected 7 days post each vaccination and subjected to antibody assays (Figure 1(A)). Compared to the pre-bleed serum, post-immunized serum (after three times DNA immunization) showed clear IgG titers against RBD, S1 and ECD, with titers of 2700, 2700 and 900 respectively. Conversely, the post-immunized serum after two protein immunization represented potent and specific serologic activities toward RBD, S1 and ECD binding with a titer of 655,100, 21,800 and 72,900 (Figure 1(B)). Hence, these results indicated that our DNA prime followed by protein boost immunization strategy successfully induced SARS-CoV-2 specific antibodies with virus-neutralizing capability.

### Functional screening of S1-specific single B cells

**Figure 2.**
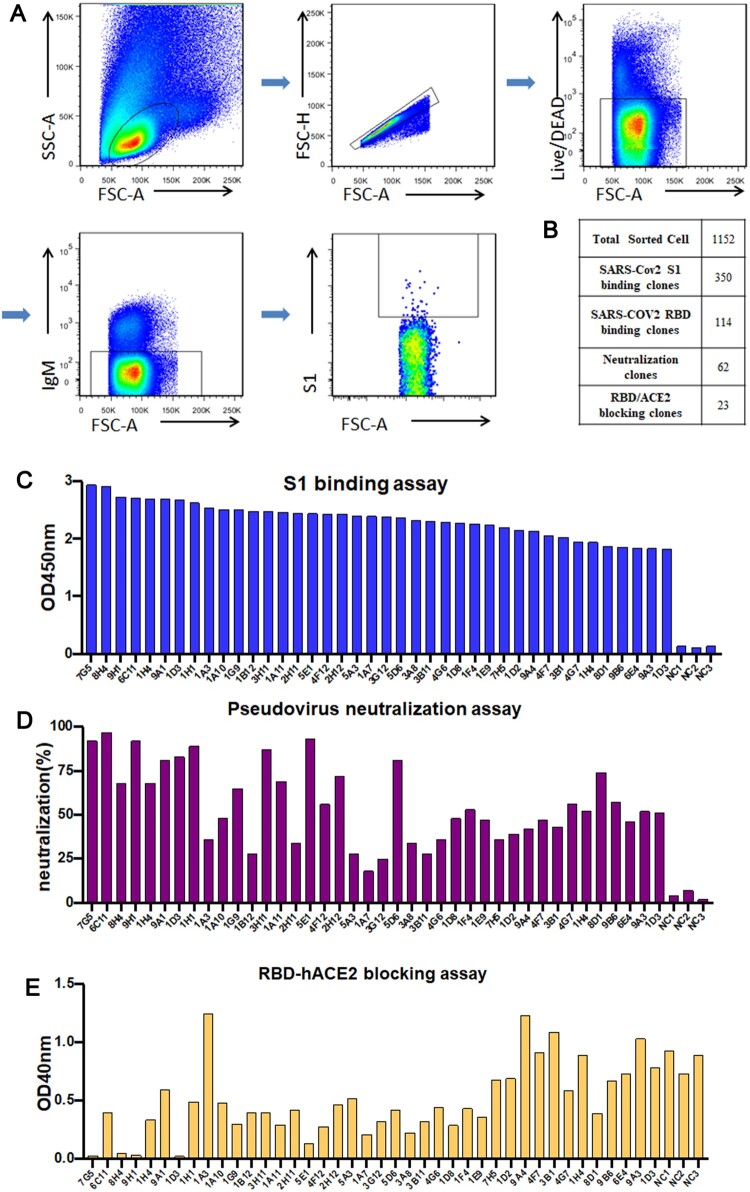
Functional screening of cultured S1-specific single rabbit B cell clones. (A) The sorting strategy for S1-specific B cells from rabbit splenocytes using FACS. (B) High-throughput functional screening of cultured B cells. The number of individually cultured B cell clones (Cultured B cell clones), B cell clones binding SARS-CoV-2 S1 protein (SARS-CoV-2 S1 binding clones), B cell clones binding the SARS-CoV-2 RBD (SARS-CoV-2 RBD binding clones), B cell clones with neutralization potential (Neutralization clones), and B cell clones with the capability of inhibiting RBD and ACE2 interaction (RBD/ACE2 blocking clones) were listed. (C) Representative ELISA screening of cultural supernatants for top 40 B cell clones that can bind to the SARS-CoV-2 S1 protein. (D) Pseudovirus neutralization assay of S1-specific B cell cultural supernatants. (E) ACE2 blocking assay of S1-specific B cell cultural supernatants.

To dissect the specificities and binding characteristics of antibody responses, the rabbit with a higher titer against S1 and RBD was subcutaneously injected with 100 μg SARS-CoV-2 S1 protein on day 64 as the final boost. 5 days after the final boost, the rabbit’s spleen was harvested (Figure 1(A)). Single S1-specific binding B cells from splenocytes were isolated by FACS using fluorescent S1 protein probes. A total of 1152 individual B cells were sorted at 1 cell per well and expanded for 7 days in the rabbit B cell complete medium. The supernatants of the cultured B cell clones were screened using ELISA. Herein, we identified 350 B cell clones that were reactive to S1, and 114 out of 350 were bind to RBD. Among S1-reactive B cell clones, 62 clones were found positive of neutralization activities and 23 blockers of ACE2-RBD interaction (Figure 2(B)). The top 40 neutralizing clones were cloned and validated for S1 binding, pseudovirus neutralization, and RBD-ACE2 inhibition (Figure 2(C–E)). Four most potent neutralizing RmAbs clones were selected for recombinant expression and further characterization.

### Binding properties of four RBD-specific RmAbs

**Figure 3.**
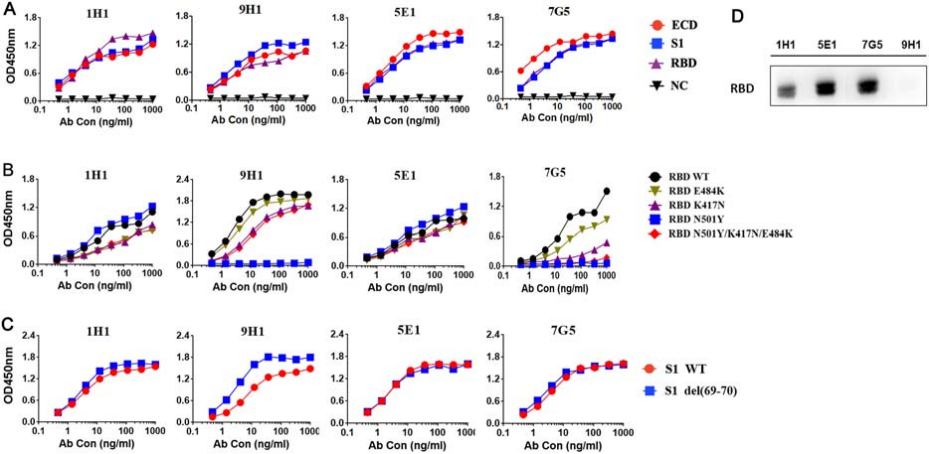
Binding of four neutralizing RmAbs, 1H1, 9H1, 7G5, and 5E1 to SARS-CoV-2 spike proteins and RBD mutants. (A) Four clones of RmAbs bind to purified SARS-CoV-2 ECD, S1 and RBD in ELISA. (B) RmAbs have different recognition of RBD and RBD mutants including RBD N501Y, RBD K417N, RBD E484K, and RBD N501Y/K417N/E481K. (C) RmAbs bind to S1 and S1 mutants S1 69-70 deletion. (D) RmAbs differentially recognize the SARS-CoV-2 RBD protein in Western Blotting.

The 4 top B cell clones (1H1, 7G5, 5E1, 9H1) were selected based on their binding epitope and neutralization potency. The naturally paired variable heavy chains (VH) and light chains (VL) were recovered from each cultured B cell clone. The VH and VL fragments were inserted into expression plasmids to express full-length heavy chain and light chain. All four mAbs potently and specific bind to the RBD, S1 and ECD protein (Figure 3(A)).

Recent SARS-CoV-2 variants emerged in United Kingdom (B.1.1.7) [48], South Africa [49], Brazil [12] and United States [7] have raised concerns on immune escape from antibody recognition. Variant B.1.1.7 has a key mutation N501Y in its spike RBD region and deletion of H69-V70 (S1 del (69-70)) on the spike. Another SARS-CoV-2 lineage, B.1.351, in South Africa (501.V2) had three altered residues in the ACE2 binding site of RBD, including K417N, E484K and N501Y. N501Y mutation leads to enhanced binding to ACE2, whereas K417N/E484K/N501Y in variant 501.V2 increases their infectivity [49]. Subsequently, to determine whether these mutations impact RmAb recognition of RBD, we first compared the antibody binding to wild-type RBD (RBD WT) with that to variants RBD N501Y, RBD K417N, RBD E484K, and RBD N501Y/K417N/E484K. 1H1 and 5E1 recognize RBD WT and RBD variants in a similar manner, but relatively weaker to RBD E484K, RBD K417N, and RBD N501Y/K417N/E484K. 9H1 binds well to RBD WT and most RBD variants except no detectable binding to RBD N501Y. 7G5 binds strongly to RBD WT and RBD E484K, weakly to RBD K417N, and fails to recognize RBD N501Y and RBD N501Y/K417N/E484K (Figure 3(B)). In addition, all four neutralizing RmAbs have a similar binding capacity for S1 WT and S1 69/70 deletion (Figure 3(C)). Thus, the data suggested that despite RBD variants were reported to be capable of immune escape, our vaccine-elicited RmAbs, 1H1 and 5E1, exhibited comparable binding strength to RBD variants. On the contrary, RBD E484K and K417N had little impact on 9H1 recognition with RBD. Moreover, our western blotting analysis revealed that the epitopes of 1H1, 5E1 and 7G5 are linear, while the epitope of 9H1 is likely conformational (Figure 3(D)).

**Figure 4.**
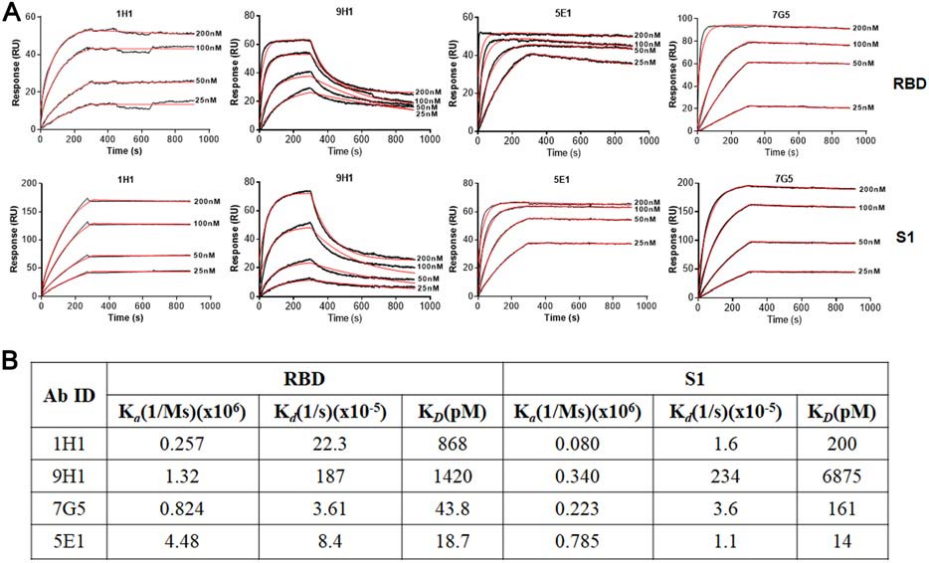
Binding kinetics of RmAbs to SARS-CoV-2 S1 and RBD proteins. (A) Surface plasmon resonance sensorgrams demonstrate the binding and dissociation kinetics of RmAbs to SARS-CoV-2 S1 and RBD proteins. Changes in resonance units (RU) over time are indicative of binding kinetics. (B) Rate constant Ka (equilibrium association constant), Kd (dissociation constant) and KD of RmAbs were determined respectively when binding to the SARS-CoV-2 S1 and RBD.

Dissociation constants (K*_D_*) of four RmAbs against the SARS-CoV-2 RBD or S1 protein were measured by surface plasmon resonance (SPR), ranging from 14.0 pM to 6975 pM (Figure 4(A, B)). 9H1 and 7G5 have 4.84-fold and 3.67-fold higher binding affinity to RBD when compared to S1 protein, respectively. 1H1 has a 4.34-fold lower binding affinity to RBD compared to S1 protein, and 5E1 has a comparable binding affinity to both S1 and RBD. The above results suggested that four rabbit mAbs potently and specific bind to both S1 and RBD.

### Neutralization potency of RmAbs against multiple SARS-CoV-2 variants

**Figure 5.**
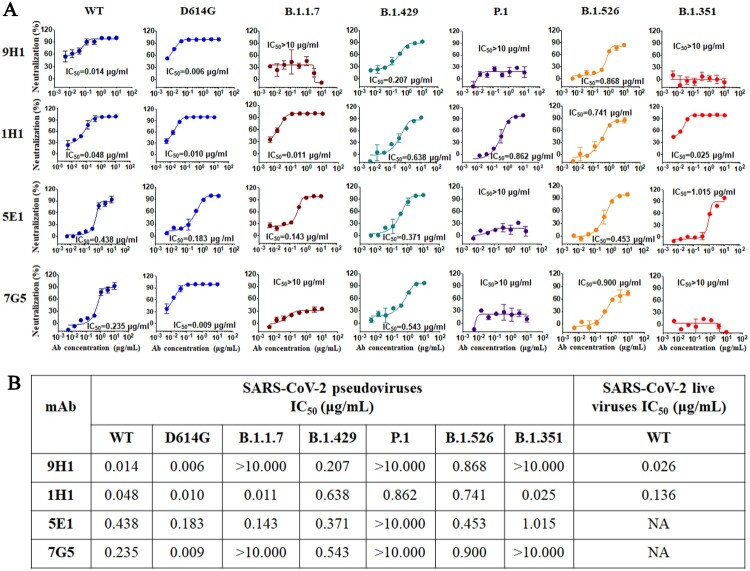
The neutralization potency of four RmAbs against SARS-CoV-2 pseudovirus variants and live viruses. (A) SARS-CoV-2 pseudovirus neutralization assay of four RmAbs (9H1, 1H1, 5E1 and 7G5) against wild type (WT) strain, the D614G variant, the B.1.1.7 variant, the B.1.429 variant, the P.1 variant, the B.1.526 variant, and the B.1.351 variant. The curve is presented by the inhibition percentage of SARS-CoV-2 pseudotyped viruses entry into host cells. (B) Neutralizing potency (IC_50_) of RmAbs was determined using either seven SARS-CoV-2 pseudotyped variants or SARS-CoV-2 live viruses.

To evaluate the neutralization potency of four rabbit mAbs, both pseudovirus assay and live viral neutralization assay were performed. We first determined the neutralization potency of four RmAbs using SARS-CoV-2 wild-type pseudotyped virus assay. The half-maximal inhibitory concentration (IC_50_) value of 9H1,1H1, 5E1, and 7G5 for wild type pseudotyped virus assay is 0.014 μg/ml, 0.048 μg/ml, 0.438 μg/ml, and 0.235 μg/ml, respectively (Figure 5(A, B)). Consistent with pseudovirus neutralization, 1H1 and 9H1 could neutralize authentic SARS-CoV-2 virus, with IC_50_ values of 0.136 and 0.026 μg/mL, respectively.

To determine whether these RmAbs could neutralize currently circulating SARS-CoV-2 variants, we then tested the neutralization potency of RmAbs against six emerging SARS-CoV-2 variants including the D614G variant, the B.1.1.7 variant, the B.1.429 variant, the P.1 variant, the B.1.526 variant, and the B.1.351 variant (Figure 5(A, B)). Specifically, we found that neutralization of D614G variant was comparable with wild-type strain by four RmAbs, in line with previous reports [50]. Consistent with ELISA data of 1H1 and 5E1 binding with RBD N501Y, the neutralization potency of 1H1 and 5E1 is similar to that of the wild type and D614G strain, while 9H1 and 7G5 which failed to bind to RBD 501Y also presented neutralization resistance of the B.1.1.7 variant. RBD L452R mutation from B.1.429 variant leads to a slightly increased neutralization resistance of these RmAbs. Since the attenuated neutralizing activity of convalescent sera against emergent variants was largely attributed to the E484K substitution [51], we then tested the other three E484K harboring variants, including P.1, B.1.526, and B.1.351. For Brazil P.1 with three RBD mutations (K417T/E484K/N501Y), only 1H1 was found to mediate neutralization in our study. Despite the neutralization potency against in the B.1.526 strain with D614G and E484K mutation has been slightly reduced by four RmAbs, all four RmAbs isolated from our study were still capable to neutralize B.1.526 variant in presence of E484K mutation. B.1.351, one variant of the greatest concern, was able to be neutralized by two vaccine-elicited RmAbs identified in our study, 1H1 and 5E1, with a comparable IC_50_ as wild-type strain. Notably, 1H1 was able to neutralize all emerging SARS-CoV-2 variants tested at microgram or sub microgram level, 5E1 could neutralize against D614G, B.1.1.7, B.1.429, and B.1.526, B.1.351 except to P.1. 9H1 and 7G5 could neutralize D614G, B.1.429 and B.1.526 variants, but not the other three variants tested.

### Epitope binning and phylogenic characterization of RmAbs

**Figure 6.**
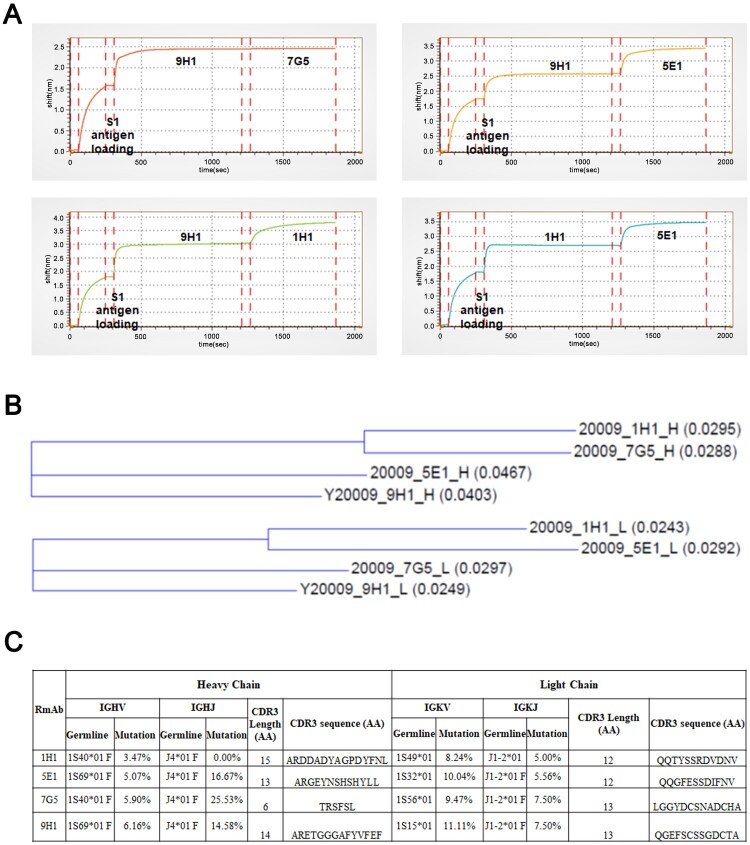
Epitope binning and sequence analysis of neutralizing RmAbs. (A) Epitope binning assay was performed to assess the pairing of mAbs when binding to the SARS-CoV-2 S1 protein. Binding intensity was indicated as the shift in nm over time. (B) Phylogenetic analysis of heavy chain (above) and light chain (below) genes for four neutralizing RmAbs using the maximum-likelihood method. Branch lengths are drawn to scale to visualize sequence diversification. (C) Mutational status of the variable regions, joining regions, and CDR3 regions of RmAb heavy chains and light chains compared to matched germline clone sequences. IGHV: Immunoglobulin heavy-chain variable region, IGHJ: Immunoglobulin heavy-chain joining region; IGKV: Immunoglobulin kappa chain variable region; IGKJ: Immunoglobulin kappa chain joining region.

To determine the epitope and sequence basis of the four neutralizing RmAbs, epitope binning and phylogenic characterization were performed. Firstly, we evaluated whether these antibodies could compete for similar epitopes using the Tandem binding model of antibody pairs by Gator. S1 protein was loaded to Gator optic probes to capture the first antibodies in each antibody pair, followed by the incubation with second RmAbs. As shown in Figure 6(A), the addition of either 1H1 or 5E1 with 9H1-loaded probes increased signal shift indicative of binding of a second antibody to S1 protein, the addition of 5E1 to 1H1 loaded probe could also increase signal shift. Therefore, the results suggest that 9H1 does not compete with either 1H1 or 5E1, and 1H1 does not compete with 5E1. On the other hand, 7G5 was not able to further elevate the signal shift on the 9H1 loaded probe, suggesting that 9H1 could compete with 7G5 for S1. Our epitope binning results suggested that 9H1, 1H1 and 5E1 recognize distinct unique epitopes on S1, while 9H1 and 7G5 are likely to share a partially overlapped epitope since 9H1 could not recognize linearized S1 protein as 7G5 did in western blotting.

Besides, to define the genetic relationship of these RmAbs, a phylogenetic tree was constructed based on variable region sequences heavy-chain and light chain genes using the maximum-likelihood method (Figure 6(B)). 1H1 and 7G5 heavy chains have less sequence diversification when compared with 9H1 and 5E1, while 1H1 and 5E1 light chains are phylogenetically closer. Finally, we conducted a detailed sequence characterization of the four neutralizing RmAbs (Figure 6(C)). The heavy chain variable region (IGHV) of 1H1 and 7G5 belong to the 1S40*01F family, and IGHV of 9H1 and 5E1 belong to the 1S69*01F family, with the same germline joining segment J4*01F. The corresponding light chain variable regions (IGKV) of RmAbs belong to 1S49*01, 1S32*01, 1S56*01, 1S15*01 families, respectively, but sharing the same joining segment J1-2*01. Somatic hypermutation (SHM) frequency of IGHV for the above four RmAbs ranges from 3.47% to 6.16%, while SHM frequency for IGHJ varies considerably from 0.00% to 25.53%. SHM frequency of IGKV for the above four RmAbs ranges slightly higher from 8.24% to 11.11%, while that of IGKJ is from 5.00% to 7.50%. Even though the length of complementarity-determining region 3 (CDR3) for 1H1, 5E1, 7G5 and 9H1 heavy chains changes widely from 5 to 15 amino acid residues (aa), the CDR3 length of light chains remains narrowly distributed between 12 and 13 aa (Figure 6(C)).

## Discussion

In this study, we described a panel of S1-binding mAbs generated from rabbit primed with a DNA vaccine encoding the SARS-CoV-2 RBD followed by S1 protein boost. Using a single B cell SMab® platform, we identified four neutralizing RBD-specific mAb for further characterization. Our vaccine-induced RBD-specific mAbs, 9H1, 7G5, 5E1 and 1H1, recognized not only RBD and S1 protein with high binding affinity, but also some recently emerging RBD variant proteins including RBD N501Y, RBD 417N, RBD E484K, and RBD N501Y/K47N/E484K. Besides, 9H1, 5E1 and 1H1 recognized distinct epitopes in the RBD domain, while 7G5 shared a partially overlapping epitope with 9H1. Consistent with their capabilities of blocking RBD-ACE2 interaction in ELISA, four RmAbs were able to neutralize SARS-CoV-2 tested by wild-type pseudovirus neutralization assay, two of which were further validated by neutralization assay of wild type live virus strain. Notably, 1H1 was also able to neutralize all six emerging SARS-CoV-2 mutant variants tested including D614G, B.1.1.7, B.1.429, P.1, B.1.526 and B.1.351 variants.

Understanding the immune response to SARS-CoV-2 is of utmost importance. A recent vaccine study reported that the rabbit animal model, RBD immunogen elicited antibody with 5-fold higher affinity to native spike antigen, compared to S1 and ectodomain (ECD) of spike protein [52]. S1 domain induced a diverse antibody repertoire recognizing the NTD and RBD regions, recombinant RBD induced high-titer antibodies focused on the RBD/receptor binding motif (RBM). Nevertheless, the S2 domain does not appear to elicit as many neutralizing antibodies as RBD or S1. These results suggested that RBD and S1 are ideal immunogens that induce high neutralizing antibodies. In our study, we used a DNA vaccine expressing RBD priming followed by S1 protein boosting, leading to the discovery of potent neutralizing RmAbs specific to RBD.

As demonstrated by ELISA and SPR assays, all 4 anti-RBD rabbit mAbs showed well recognition of three spike protein-related antigens, including RBD, S1 and ECD, despite only S1 and RBD were used for immunization. It is noteworthy that 1H1 has a higher affinity to S1 protein than RBD protein, 9H1 and 7G5 were found to bind RBD with relatively higher affinity if compared to their binding to S1 protein, while 5E1 had a comparable binding affinity for both S1 and RBD. Additionally, the epitope binning assay revealed that 9H1, 5E1 and 1H1 recognize distinct epitopes in the RBD domain, while 7G5 shares likely an overlapping epitope with 9H1. Hence, results propose that these rabbit mAbs recognize diverse epitopes on RBD regions of SARS-CoV-2 spike protein. The data is also consistent with our heterologous prime-boost vaccination strategy, in which the priming DNA vaccine expressing RBD antigen firstly stimulated naïve B cells to generate RBD-specific mAbs and further underwent affinity maturation and somatic hypermutation to give rise to mature antibodies with high affinity and broad diversity. The generally better recognition of RBD than S1 of our RmAbs also reflects the switching of the boosting immunogen to RBD in the prime-boost immunization strategy. Boosting with RBD promoted a more focused immune response and antibody maturation to RBD-specific repertoire.

The best binding affinity does not necessarily confer the highest neutralization capability. 9H1 and 1H1 had potent neutralization capability as demonstrated by both SARS-CoV-2 wild-type pseudovirus neutralization assay and authentic virus neutralization assay. The most potent neutralizing RmAb, 9H1, showed 34.38-fold weaker binding affinity to S1 and 1.63-fold lower binding affinity of RBD when compared to 1H1, whereas 9H1 has 5.23-fold increased neutralizing potency than 1H1 as revealed by authentic virus neutralization assay. A plausible explanation is that the conformational epitope of 9H1 may be better exposed on the trimeric spike protein of SARS-CoV-2 native virions compared to that on S1 or RBD protein. Alternatively, 9H1 could more effectively promote binding avidity to trimeric spike proteins on the virion surface. Nevertheless, consistent with the relative weaker binding affinity with wild-type RBD, 9H1 was found to have a relative limited neutralization breadth, which failed to neutralize the emerging variants, including B.1.1.7, P.1 and B.1.351. The fine epitope of 9H1 and 1H1 remains yet to be defined.

Developing new vaccines to elicit broadly neutralizing antibodies and identifying of therapeutic monoclonal antibodies capable to neutralize circulating SARS-CoV-2 variants are essential to resolve the ongoing COVID-19 pandemic. In our study, in addition to SARS-CoV-2 wild type strain, we tested a panel of emerging SARS-CoV-2 variants using VSV-based SARS-CoV-2 pseudovirus neutralization assay, including the D614G variant, the United Kingdom B.1.1.7 variant, the California B.1.429 variant, the Brazil P.1 variant, the New York B.1.526 variant, and the Brazil B.1.351 variant. Accumulating evidence demonstrated that several key mutations within RBD region could confer potent neutralization escape not only in COVID-19 convalescent plasma [53], but also in vaccinee sera elicited by mRNA vaccination [54], inactivated virus vaccine, or protein-based vaccine [55]. Notably, in our study, 1H1 was able to neutralize all 6 emerging variants tested including B.1.351 and P.1, two VOCs refractory to most of well-characterized mAbs with EUA approval [51, 56]. Moreover, 5E1 could neutralize 5 out of 6 variants tested but not P.1. It is interesting to determine whether such broad neutralization of RmAbs identified in our study is resulted from heterologous DNA prime protein boost vaccination strategy used in our study [41]. Additionally, our result was also assuring that despite of RBD mutations presented in recent circulating SARS-CoV-2 isolates, our RmAbs induced by vaccines formulated from wild-type strain in our study were capable of neutralize against most of emerging variants.

To the best of our knowledge, this is the first panel of RmAbs that neutralize the SARS-CoV-2 wild type and the variants strains. A list of rabbit mAbs has been approved by FDA for diagnostics and therapeutics, encouraging the great clinical potentials of rabbit mAbs. Different from mice, rabbits have a unique B cell ontogeny that generates a highly diverse antibody repertoire. Furthermore, rabbit B cells undergo sophisticated affinity maturation processes which include both somatic hypermutation and gene conversion. Additionally, being genetically diverse and evolutionarily distinct from mice, rabbit antibodies can be generated against unique epitopes that might be non-immunogenic in mice. This allows the generation of rabbit mAbs with superior sensitivity, affinity and specificity if compared to mouse mAbs. Indeed, in our study, we identified 4 RmAbs with diverse epitope recognition, high affinity, and broad neutralizing activity. Four isolated RmAbs were able to potently neutralize SARS-CoV-2 and most of the emerging SARS-CoV-2 variants, suggesting that RmAbs might target at certain unique epitopes which were cross-reactive with multiple SARS-CoV-2 VOCs including P.1 and B.1.351. In contrast, the P.1 and B.1.351 were refractory to most of EUA approved RBD-specific mAbs [51, 56], which were isolated from convalescent individuals or humanized mice.

Compared to the conventional RmAb discovery approach using hybridoma technology, our strategy of using a high-throughput single-B-cell cloning approach is efficient and robust to identify functional rabbit mAbs. Taking advantage of the single B cell SMab® platform, we were able to clone and express the recombinant mAbs within 2 weeks. Our approach enables the accelerated discovery and identification of novel mAbs specific to newly emerged pathogens, resulting in a fast and effective response to challenging clinical needs.

Our study provides promising diagnostic and therapeutic measures against the SARS-CoV-2 but needs further experimental evidence and validation. Although our study identified a panel of rabbit mAbs with potent *in vitro* neutralization activities, the prophylactic or therapeutic potential of these RmAbs has not been fully validated in animal infection models. The fine epitopes of these RmAbs are yet to be defined for specific mechanisms of action in virus neutralization. Moreover, the humanization of these promising RmAbs is needed to better evaluate their therapeutic potentials.

In summary, we identified four potent neutralizing RmAbs specific to the SARS-CoV-2 spike RBD region from a rabbit immunized with DNA vaccine prime followed by a boost of protein vaccine. These anti-RBD RmAbs induced by the heterologous prime-boost vaccine had broad and potent neutralizing activity against SARS-CoV-2 and most of the currently global circulating SARS-CoV-2 variants. Our newly identified RmAbs may proffer a novel means for versatile, cost-effective therapeutics and point-of-care diagnosis.
